# Morphological Features and Immunohistochemical Profiling of Male Breast Gynaecomastia; A Large Tissue Microarray Study

**DOI:** 10.3389/fonc.2022.875839

**Published:** 2022-06-23

**Authors:** Prakruthi Prasad, Aneliese Bennett, Val Speirs, Abeer M. Shaaban

**Affiliations:** ^1^ School of Medicine, University of Sheffield, Sheffield, United Kingdom; ^2^ Cellular Pathology, Mid Yorkshire Hospital National Health Service (NHS) Trust, Dewsbury, United Kingdom; ^3^ School of Medicine, Medical Sciences and Nutrition, Institute of Medical Sciences, University of Aberdeen, Aberdeen, United Kingdom; ^4^ Queen Elizabeth Hospital Birmingham and Cancer and Genomic Sciences, University of Birmingham, Birmingham, United Kingdom

**Keywords:** gynaecomastia, male breast, immunohistochemistry, breast cancer, male breast cancer

## Abstract

**Introduction:**

Gynaecomastia is the commonest male breast condition accounting for approximately 85% of male breast lesions. There is minimal information on the immunohistochemical profile of gynaecomastia. We aimed to comprehensively profile a large series of gynaecomastia samples for putative mammary diagnostic, predictive and prognostic markers.

**Methods:**

A total of 156 samples, were histologically reviewed, assembled onto tissue microarrays, and stained for oestrogen receptors (ERα, ERβ1, ERß2), progesterone receptors (total PR, PRα), androgen receptor (AR), basal & luminal cytokeratins (CK5/6, CK14, CK8/18) and the proliferation marker Ki67. Relevant cut offs for marker positivity were defined based on existing literature: AR (10%), ERα and PR (Allred score >3/8), ERß (10% and 20%), cytokeratins (10%) and Ki67 (10% and 20%).

**Results:**

108 samples from 86 patients aged 13-75 years were available for immunohistochemical assessment. 73.1% of the lesions were AR positive, compared to 99% for ERα and 100% for both ERß1 and ERß2. 98% of samples were positive for total PR and 97.1% for PRα. 69.8% expressed CK5/6 whilst 57% were CK14 positive. A tri-layered pattern of cytokeratin expression was also observed. Ki67 positivity was low with 17.1% and 6.7% classified as Ki67 positive using 10% and 20% cut off values respectively. A significant negative correlation was found between ERα expression and patient age (rs = -0.221, p=0.023). Bivariate correlations were produced, and comparisons made with previously published data regarding the immunohistochemical status in normal female breast tissue, proliferative and neoplastic breast diseases of the female and male breast.

**Conclusions:**

Hormone receptors, including oestrogen receptor α and ß isoforms as well as androgen receptors were abundantly expressed within the intraductal luminal hyperplastic epithelium in gynaecomastia supporting the hormonal role in the pathogenesis and treatment. ERα, ERβ1 and ERβ2 were expressed in a higher proportion of cells compared with their expression in the female breast benign lesions which further characterises gynaecomastia biology. The identification of a low Ki67 proliferative index and the mixed cytokeratin profile in gynaecomastia differentiates this benign condition from male breast cancer. Therefore, Ki67 and cytokeratins can help in the differential diagnosis from histological mimics in the routine diagnostic work up.

## Introduction

Gynaecomastia is a benign, non-neoplastic proliferative condition and is the most common lesion in the male breast. Pseudogynaecomastia, or lipomastia, refers to the accumulation of adipose tissue in the male breast without glandular hyperplasia (1). True gynaecomastia can be differentiated from pseudogynaecomastia by the presence of dense subareolar ductal tissue and fibrosis. There are several aetiological factors for true gynaecomastia including physiological causes, such as neonatal, adolescent, and elderly gynaecomastia, resulting from an imbalance between oestrogen stimulation and androgen inhibition (2). Pathological causes of gynaecomastia include Klinefelter’s syndrome, where hypogonadism causes an increased oestradiol-to-testosterone ratio leading to the genesis of gynaecomastia (3). Drugs, obesity, and relative oestrogen excess (e.g., due to liver cirrhosis or prostate cancer) are amongst other causes of gynaecomastia. However, most gynaecomastia cases are regarded as idiopathic with no apparent cause. There is no proven link between gynaecomastia and the development of male breast cancer (4, 5).

The histological features of gynaecomastia are well documented. Microscopically, the early stage of gynaecomastia (florid phase) is characterised by proliferative branching ducts with epithelial and stromal hyperplasia, and the late stage (fibrous phase) is characterised by collagenous stroma, less epithelial proliferation, and decreased vascularity (4).

Little is known about the immunohistochemical profile of male gynaecomastia and how it compares to the female breast due to the lack of large studies to date analysing the expression of putative diagnostic and prognostic markers within this lesion. Therefore, the aim of this study was to analyze the expression of several relevant proteins, including hormone receptors, cytokeratins (luminal and basal types) and proliferation markers in a large, well characterised cohort of surgically resected gynaecomastia specimens.

## Materials and Methods

Ethical approval was sought and granted from the Leeds (East) Research Ethics Committee (reference number 06/Q1205/156). Male patients who had undergone surgical excision for gynaecomastia at the Leeds Teaching Hospitals NHS Trust, during the period between 2000 to early 2011 were identified from the pathology database. Histological sections of those patients were collected and reviewed by two investigators (AB, AMS) to confirm the diagnosis, identify the morphological appearances, classify into early or late stage, and mark representative areas for tissue microarray (TMA) construction. Comprehensive clinical details were collected including patient age, presentation, laterality, history of previous medications, history of previous cancer and family history where available.

### Full Face Immunohistochemistry

Prior to the construction of TMAs, representative full sections for 10% of the gynaecomastia cases studied were randomly selected and immunohistochemically stained for the complete panel to assess for staining heterogeneity. Staining was overall uniform across sections and tissue microarrays were deemed a suitable method for analysing the large sample numbers.

### TMA Construction

Representative tissue cores from the donor blocks were assembled onto tissue microarrays.

Tissue microarrays (TMAs) were constructed using a manual tissue arrayer (Beecher Instruments, Inc., Sun Prairie, W1, USA) using representative 0.6mm tissue cores from representative marked areas. For each case, 4 cores, arranged in duplicates, were included. TMAs were sectioned at 3 microns and stained with the panel of immunohistochemical biomarkers. Cores of various other human tissues including female breast carcinoma, prostatic carcinoma, normal prostate, endometrium and appendix were assembled in the TMAs in an orderly fashion to serve as positive controls and also for orientation as previously described (6). For AR, ERα, total PR, CK5/6, CK8/18, CK14 and Ki67 optimised immunohistochemical protocols on Dako Autostainer Link 48 routinely used within the Leeds Teaching Hospitals NHS Trust diagnostic histopathology department and approved by United Kingdom National External Quality Assurance Scheme (NEQAS) were followed in accordance with standard operating procedures.

The initial immunohistochemical stage incorporated slide pre-treatment, whereby the formalin-fixed paraffin embedded tissue sections underwent deparaffinisation, hydration and heat-induced epitope retrieval (HIER) by immersion in the automated DAKO Envision Flex High pH Link solution system. During this timeframe refrigerated (4°C), commercial concentrated monoclonal mouse antibodies raised against human AR, ERα, total PR, CK5/6, CK8/18, CK14 and Ki67 tissue proteins were diluted in accordance with pre-determined, optimised dilution ratios (**Table 1**) and placed within defined Dako reagent bottles. These diluted primary antibodies and ready-to-use reagents supplied within the DAKO Envision HRP/DAB+ Flex Plus High detection kit were placed within the Dako reagent racks. After completion of HIER, slides were washed in Envision Flex wash buffer. Slides and reagent racks were loaded onto the DAKO Autostainer Link 48 for integrated, automated section staining for tissue antigen visualisation using the Dako Autostainer Link 48, following manufacturer’s instructions.

**Table 1:** Details of the studied antibodies.

Antibody	Supplier	Clone	Host	Dilution	Positive control	Localisation
AR	Dako	M3562	Mouse	1:800	Prostate	Nuclear
ERα	Novocastra	6F11	Mouse	1:200	Breast carcinoma/ Endometrium	Nuclear
Total PR	Dako	PgR 636	Mouse	1:800	Breast carcinoma/ Endometrium	Nuclear
PRα	Novocastra	PGR/312	Mouse	Pre-diluted	Breast carcinoma/ Endometrium	Nuclear
ERβ1	Serotec	PPG5/10	Mouse	1:2	Breast carcinoma/ Endometrium	Nuclear
ERβ2	Serotec	57/3	Mouse	1:100		Nuclear
CK5/6	Dako	D5/16	Mouse	1:100	Prostate	Cytoplasmic
CK14	Novocastra	B4	Mouse	1:100	Prostate	Cytoplasmic
CK8/18	Novocastra	LL002 5D3	Mouse	1:200	Appendix	Cytoplasmic
Ki67	Dako	MIB-1	Mouse	1:300	Tonsil	Nuclear

For ERβ1, ERβ2 and PRα a manual staining protocol using a Shandon sequenza rack was performed as previously described (7). Positive controls were included in each batch of staining (**Table 1**). All sections were counterstained with Mayer’s haematoxylin and Scott’s tap water substitute.

The immunohistochemically stained TMA slides were scanned and assessed using Aperio ScanScope XT Scanner and Aperio ImageScope Software (Aperio Technologies) for manual computer-based tissue core analysis at high resolution. For all biomarkers, the percentage of positively stained glandular luminal cells was semi-quantified. For the hormonal biomarkers, the staining intensity was also evaluated and recorded. Relevant cut offs for marker positivity were chosen based on those most used in existing literature: AR (10%) (8), ERα (Allred score >3/8) (9), ERß (10% and 20%) (7, 10, 11), PR (Allred score >3/8) (9),CKs (10%) (12), and Ki67 (10% and 20%) (13).

### Statistical Analysis

Data analysis was done using IBM SPSS Statistics version 27 programme. The percentages of samples positive for each receptor and combination of receptors were calculated. Correlations were two sided and considered as statistically significant when p ≤0.05 and highly significant when p <0.001. Pearson correlations were calculated between biomarkers, and between biomarkers and age. The non-parametric Mann-Whitney U test was used to assess the relation between age as a continuous variable and the presence/absence of hyperplasia.

## Results

### Patient Characteristics

A total of 118 patients fulfilled the inclusion criteria, of whom 38 had bilateral gynaecomastia. For the latter, tissues from each breast were reviewed and representative blocks selected. Cases diagnosed as gynaecomastia on core biopsy only without a surgical specimen over the 11 years search period were excluded.

Following histological review, surgical specimens comprising predominantly fatty tissue with little/no glandular elements were excluded from the immunohistochemical part of the study. Therefore, a total of 156 gynaecomastia samples were used for TMA construction and immunohistochemical analysis.

After excluding non-representative and missing cores, immunohistochemical data was available for 108 samples from 86 patients. 24 of these patients had bilateral samples taken. 48 samples were taken from the right breast, 59 samples were taken from the left breast. Laterality was not known for one sample. Patients’ age ranged from 13-75 years, with a mean of 28.07 years ( ± SD:14.97).

Data of previous therapies were not available for 50% of patients. 30.6% had not received any previous therapies. 5.6% had a history of the selective oestrogen receptor modulator Tamoxifen, 3.7% had received Danazol therapy. One patient had Docetaxel therapy and another 2.8% received previous Casodex therapy, both of which were indicated for metastatic prostate cancer. Only one patient had a family history of breast cancer. 5.6% of patients had a history of illicit drug use (cannabis and cocaine) and one patient had previously used steroid body building supplements.

### Morphological Appearances

**Figure 1:**
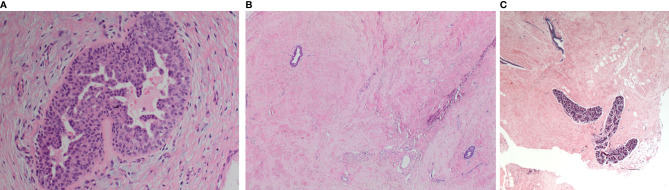
Morphological appearances of the male breast gynaecomastia cases. **(A)** Florid gynaecomastia showing a mammary duct with micropapillary epithelial hyperplasia. The surrounding stroma is cellular and oedematous. **(B)** Late (fibrous) gynaecomastia: mammary ducts with minimal hyperplasia within a fibrosed stroma. **(C)** Well-developed mammary lobules were occasionally observed.

All cases were examined for the characteristic morphological appearances of gynaecomastia on H&E sections. A total of 93 samples (86.1%) showed active (early) gynaecomastia with associated epithelial hyperplasia. There was no significant correlation between age and the histological appearance of gynaecomastia (Mann-Whitney U, p=0.306). Active gynaecomastia was characterised by hyperplastic branching ducts within epithelial proliferation and periductal stromal oedema (**Figure 1A**). The stroma was generally cellular with increased vascularity. Patchy lymphocytic infiltrate, inspissated secretions, pseudo-angiomatous stromal hyperplasia (PASH) and hyalinisation were occasionally noted. Morphologically distinct latent phase gynaecomastia was observed in the remaining smaller proportion of gynaecomastia cases. This latter phase of dormancy was characterised by stromal fibrosis and sparse glandular elements (**Figure 1B**). No periductal oedema or prominent stromal vascularity was noted. While mammary lobules were not generally described as a feature of the male breast, examples of mammary lobules resembling those identified in the female breast were seen in a small proportion of cases (**Figure 1C**).

### Hormone Receptor Expression in Gynaecomastia

**Figure 2:**
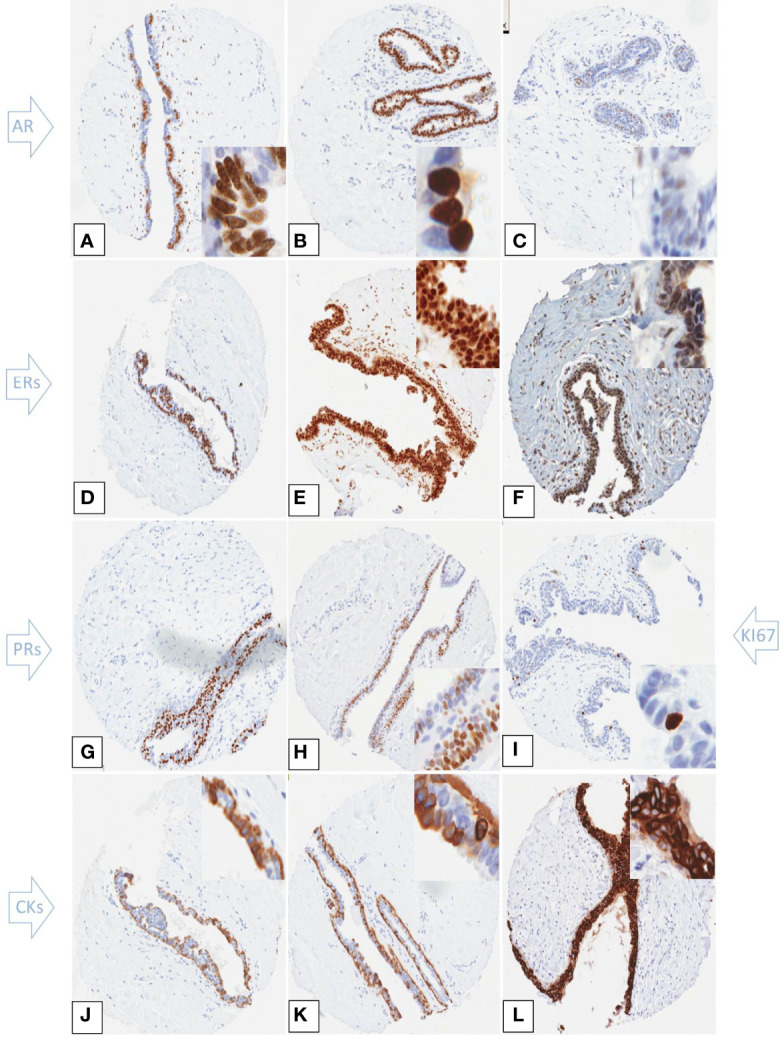
Immunohistochemical expression of hormone receptors, Ki67 and cytokeratins in tissue microarray cores of gynaecomastia. Insets are higher power views of the cellular expression. **(A–C)** Androgen receptor staining showing strong patchy **(A)** and uniform **(B)** nuclear staining. **(C)** shows occasional AR weakly stained cells. **(D)** ERα strong nuclear staining in the majority of nuclei of the hyperplastic ducts. ERβ1 **(E)** and ERβ2 **(F)** showed strong nuclear expression within both the epithelial and stromal cells. Total PR **(G)** and PRα **(H)** exhibited strong nuclear staining in most of epithelial cell nuclei. The Ki67 proliferation index was low within the hyperplasic ducts **(I)**. CK5/6 **(J)** and CK14 **(K)** were positive within the mammary ducts that showed a tri-layered pattern of cytokeratin staining. The inner and outer layers were CK5/6 and CK14 positive while the middle layer was negative. CK8/18 showed strong and uniform membranous staining of the epithelial cells **(L)**.

All hormone receptors analyzed, including ERß and PR isoforms, were highly expressed in gynaecomastia lesions (**Table 2**). Percentages were calculated from the core samples that had available data. For ERß isoforms, both cut off values used in the literature (10% and 20%)^7^ yielded identical results. Luminal cytokeratins were positive in all cases examined whereas basal cytokeratins CK5/6 and CK14 were expressed in 69.8% and 57% of the cases respectively (**Figures 2A–L**). The tri-layered pattern of basal cytokeratin expression enclosing a luminal layer has been identified in a patchy fashion (**Figures 2J, K**). The Ki67 proliferation index was low (**Table 3**).

**Table 2:** Details of immunohistochemical expression of the studied markers in gynaecomastia lesions.

Marker	AR	ERα	ERβ1	ERβ2	PR	PRα
Number	108	105	105	100	100	104
Mean % ( ± SD)	53.52 ( ± 38.246)	79.48 ( ± 18.604)	98.76 ( ± 3.847)	91.60 ( ± 8.873)	56.70 ( ± 23.881)	56.44 ( ± 21.885)
Minimum	0	0	80	50	0	0
Maximum	100	100	100	100	95	90
Percentage Positivity	73.1	99	100	100	98	97
**Staining intensity**						
Number	108	105	105	100	100	104
Negative	26 (24.1)	2 (1.9)	1 (0.95)	0	2 (2)	3 (2.9)
Weak	10 (9.3)	21 (20)	1 (0.95)	1 (1)	16 (16)	9 (8.7)
Moderate	45 (41.7)	55 (52.4)	94 (89.5)	96 (96)	68 (68)	87 (83.7)
Strong	27 (25)	28 (26.7)	10 (9.5)	3 (3)	14 (14)	5 (4.8)
**Cytoplasmic positivity**						
Positive	NA	NA	105	99	NA	27
Negative	NA	NA	0	1	NA	73
**Stromal positivity**						
Positive	57	4	103	100	0	0
Negative	51	104	3	8	108	104

N/A, not applicable.

**Table 3:** Luminal and basal cytokeratins and Ki67 expression in gynaecomastia lesions.

Marker	CK5/6	CK14	CK8/18	Ki67
Number	106	107	108	105
Mean ( ± SD)	19.01 ( ± 17.934)	15.14 ( ± 16.143)	98.935 ( ± 4.689)	4.371 ( ± 6.420)
Percentage positive	69.8	57	100	17.1 (10% cut-off) 6.7% (20% cut-off)

10.5% of samples showed 100% ERα expression. 23.1% of samples showed no (0%) AR expression and 4.6% showed 100% AR expression. The majority of samples (41.7%) showed moderate staining of AR in the luminal cells.

When co-expression of hormone receptors was analyses, 66.7% of samples were positive for AR, ERα and PR. 68.5% were positive for AR, ERß1 and PR, compared to 66.7% for AR, ERß2 and PR (**Table 4**).

**Table 4:** Number and proportion of samples positive for combination of hormone receptors (n = 108).

MarkerN (%)	AR	ERα	ERß1	ERß2	PR	PRα
AR		76 (70.4)	77 (71.3)	75 (69.4)	72 (66.7)	74 (68.5)
ERα			102 (94.4)	97 (89.8)	98 (90.7)	98 (90.7)
ERß1				100 (92.6)	96 (88.9)	100 (92.6)
ERß2					91 (84.3)	96 (88.9)
PR						93 (86.1)
PRα						

The correlations between age and expression of each biomarker were also studied. There was a significant negative correlation between ERα expression and age (rs= -0.221 p=0.023); a feature opposite to that seen in the female breast. ERα was the only biomarker to show a significant correlation with age. ERα also showed a highly significant positive correlation with AR (0.499, p<0.001), PR (0.305, p=0.002), PRα (0.340, p<0.001), and significantly correlated with CK8/18 (0.219, p=0.025). High ERß1 significantly correlated with lower CK14 expression (-0.224, p=0.025). ERß2 showed a significant positive correlation with PRα (0.272, p=0.006). Highly significant correlations were noted between AR and both PR (0.281, p=0.005) and PRα (0.340, p<0.001). Ki67 showed no significant correlation with any of the studied markers. The correlations between percentage expression of the studied biomarkers are shown in **Table 5**.

**Table 5:** Correlation between biomarkers’ expression.

VariableCorrelation (p value)	AR	ERα	ERß1	ERß2	PR	PRα	CK5/6	CK14	CK8/18	Ki67
AR		0.499 (<0.001)**	0.034 (0.731)	0.051 (0.613)	0.281 (0.005)*	0.340 (<0.001)**	-0.167 (0.091)	0.115 (0.246)	0.181 (0.061)	-0.105 (0.285)
ERα			0.180 (0.068)	0.098 (0.338)	0.305 (0.002)*	0.315 (0.001)**	-0.098 (0.328)	-0.099 (0.327)	0.219 (0.025)*	-0.068 (0.495)
ERß1				0.102 (0.312)	0.197 (0.051)	-0.009 (0.929)	0.077 (0.447)	-0.224 (0.025)*	0.008 (0.932)	0.047 (0.640)
ERß2					-0.030 (0.772)	0.272 (0.006) *	0.138 (0.181)	-0.003 (0.977)	0.103 (0.306)	0.041 (0.688)
PR						0.537 (<0.001)**	-0.059 (0.565)	-0.013 (0.901)	0.053 (0.604)	0.084 (0.406)
PRα							-0.035 (0.727)	-0.067 (0.505)	0.085 (0.389)	-0.123 (0.217)
CK5/6								0.283 (0.005)*	-0.093 (0.350)	-0.002 (0.980)
CK14									-0.157 (0.113)	0.005 (0.964)
CK8/18					.					-0.147 (0.134)
Ki67										

*Significant p value (<0.05).

**Highly significant p value (<0.001). Bold values are significant or highly significant values.

## Discussion

Research into male breast lesions has been limited compared with the female breast. Previous, generally small scale, studies on immunohistochemistry of gynaecomastia yielded conflicting results. Here, we detail the immunohistochemical profile of a large series of male breast gynaecomastia lesions represented on TMAs and the association between different protein markers.

Hormones have long been implicated in the pathogenesis of gynaecomastia and endocrine treatment, including androgens, anti-oestrogens, and aromatase inhibitors are currently in use for the medical management of those lesions (14, 15). When gynaecomastia persists after a period of reassurance and observation and/or discontinuation of exposure, Tamoxifen (a selective Estrogen receptor modulator, SERM) is the first medical treatment option (14). Tamoxifen is known to reduce pain and size of gynaecomastia. Examples of lesions that have completely resolved following Tamoxifen treatment have also been reported (16). Danazol, a weak androgen, has been also used but is less effective than Tamoxifen and may indeed exacerbate gynaecomastia due to its conversion to oestrogen and/or the resultant weight gain (17).

We report a predominance of various types of nuclear steroid receptors in gynaecomastia with over two thirds of the lesions expressing androgen receptor. A previous small study reported 80% AR positivity in those lesions (18). An earlier study reported AR positivity in 100% of gynaecomastia samples, compared to 87% positivity in male breast carcinoma (19). A strong correlation between ERα and AR was noted in this study (0.499, p<0.001), which is similar to a previously recorded close association noted between the two markers (p<0.01) (19).

AR immunoreactivity has been shown to range between 38% and 81% in MBC (20) and the presence of it has shown to correlate with better overall survival in MBC (21). The AR expression shown in this study supports the use of non-aromatising androgen therapies, such as dihydrotestosterone, which has shown good response rates in patients with prolonged pubertal gynaecomastia (22).

One intriguing finding in our study is the inverse correlation between ERα expression and age with a decrease in its expression with the increase in patient’s age. To our knowledge, this is a novel finding and is different from the female breast where ERα expression has been shown to increase in mammary epithelium of post-menopausal women, likely a response to decreased circulating levels of oestrogen and increased sensitivity of the receptors (23, 24). The reason for this finding in the male breast awaits further studies but may be due to the relative increase of serum estradiol relative to androgen in elderly men or disturbances in the local breast tissue response to estrogen due to the decreased inhibitory effect of androgen as a result of aging. **Table 6** summarises the expression of hormone receptors in non-neoplastic and malignant breast lesions in men and women as reported in the literature.

**Table 6:** Comparative expression of hormone receptors in gynecomastia and male and female breast cancer.

Condition	% expression of hormone receptor (range)						References
	AR	ERα	ERβ1	ERβ2	PR	PRα	
Female normal	20 (5-31)	20-28.22	88.5- 97	78	29 (10-30)	10-20	(8, 10, 23, 25–28)
Gynaecomastia	54	79	99	92	57	56	Current study
Female epithelial hyperplasia	n/a	30.27 - 57	67.50 - 80.50	46.7	70	4.3 Allred score	(10, 23, 27, 29)
Male breast cancer	95	80	61	81	71	76	(21)
Female breast cancer	92	68	92	77	72	48	(21)

N/A, not applicable.

Within our current study, 99% of samples were deemed ERα positive. High percentages of ERα positivity have been documented in both gynaecomastia and MBC at 100% and 87% expression respectively (30). Cases of MBC are often found to have a high percentage of ERα expression compared to female breast cancer (83% and 68% respectively) (30).

Another novel finding is the high expression of ERß isoforms, ERß1 and ERß2, in all the gynaecomastia lesions examined. In addition to the localisation in the lesional epithelial cells, ERß isoforms were also expressed in the stromal cells suggesting a role of ERß in the pathogenesis of gynaecomastia. Nicoletti et al. confirmed high levels of ERß RNA expression in primary cultured cells from 50 examples of male pubertal gynaecomastia including in stromal cells (31). They concluded that the data support a role of ERß in the pathophysiology of pubertal gynaecomastia. However, that same study noted a significant inverse correlation between AR and ERα (p<0.01) in carcinoma (31). An inverse correlation was also noted between AR and PR (p<0.01) in carcinoma cases. In contrast, our study noted a significant positive correlation between AR and PR (0.281, p=0.005), indicating that the relationship between biomarkers within gynaecomastia is distinctly different from malignant male breast lesions.

Little information is available on the basal and luminal cytokeratin expression in gynaecomastia. Here, we confirm the previously reported tri-layered pattern of expression of outer and inner positive basal cells enclosing a middle luminal layer. Within this tri-layer epithelium of gynaecomastia lesions, CK5/6 and CK14 are commonly expressed, 67% and 21% respectively in the inner luminal layer, 6% and 1% respectively in the intermediate luminal layer, and 84% and 99% respectively in the outer myoepithelial layer^2^. Our study showed similar results of 68.9% expression of CK5/6 and 57% expression of CK14 within glandular epithelium. Basal cytokeratins therefore can be used in the diagnostic setting to confirm hyperplasia. In the female usual ductal hyperplasia, the expression of basal cytokeratins occurs in a heterogeneous/mosaic fashion with positive basal like cells admixed with negative luminal cells (4). Awareness of the different patterns of the immunohistochemical expression of CK5/6 and CK14 in the male and female hyperplastic lesions is important to avoid mistaking the tri-layered pattern in male breast for Pagetoid spread/atypia. It is of note that, in male breast cancer, a basal profile is extremely uncommon (5, 32, 33).

In conclusion, we report on the morphological and immunohistochemical features of a large cohort of male breast gynaecomastia lesions received in a large single institution over 11 year period. The majority of the lesions showed hyperplasia, exhibited high levels of oestrogen and progesterone receptors as well as androgen receptors. We confirm, for the first time, the abundance of ERß isoforms, both in the epithelial and stromal elements, and the negative association between ERα and age. The findings suggest a major role of hormonal factors in the pathogenesis of gynaecomastia and the value of anti-hormonal therapy in the medical management of this common condition. Using hormone receptors basal cytokeratins and ki67 can help differentiate florid examples of hyperplastic male breast lesions from the malignant mimics.

## Data Availability Statement

The original contributions presented in the study are included in the article/supplementary material. Further inquiries can be directed to the corresponding author.

## Ethics Statement

The studies involving human participants were reviewed and approved by Leeds (East) Research Ethics Committee (reference number 06/Q1205/156). Written informed consent from the participants’ legal guardian/next of kin was not required to participate in this study in accordance with the national legislation and the institutional requirements.

## Author Contributions

PP: analyzed the data and wrote the first draft; AB: performed the laboratory work, collected patient data, analyzed the data and contributed to writing up; VS: ethical approval, contributed to writing up; AS: conceived the idea, supervised experimental work, oversaw writing up. All authors approved the final manuscript.

## Funding

AS is supported by Birmingham Cancer Research UK Centre grant C17422/A25154.

## Conflict of Interest

The authors declare that the research was conducted in the absence of any commercial or financial relationships that could be construed as a potential conflict of interest.

## Publisher’s Note

All claims expressed in this article are solely those of the authors and do not necessarily represent those of their affiliated organizations, or those of the publisher, the editors and the reviewers. Any product that may be evaluated in this article, or claim that may be made by its manufacturer, is not guaranteed or endorsed by the publisher.
